# Supplementary material for: A sensitive and specific genetically-encoded potassium ion biosensor for in vivo applications across the tree of life
Source: PLoS Biol. 2022 Sep 6;20(9):e3001772. doi: 10.1371/journal.pbio.3001772 (PMC9481166; doi:10.1371/journal.pbio.3001772)
Supplement: S1 Table — (DOCX) [file pbio.3001772.s001.docx]

**Table S1. X-ray data collection and refinement statistics.**

| Crystal | GINKO1 |
| --- | --- |
| **Data collection** | |
| Spacegroup | P1 |
| a, b, c (Å) | 46.8, 49.3, 83.7 |
| α, β, γ (°) | 89.96, 89.97, 80.95 |
| Resolution (Å) | 42.08-1.85 (1.92 – 1.85) |
| *R*_merge_ | 0.080 (0.834) |
| *R*_meas_ | 0.109 (1.13) |
| Multiplicity | 1.9 (1.9) |
| CC(1/2) | 0.993 (0.591) |
| CC* | 0.998 (0.862) |
| I/σ(I) | 6.3 (1.1) |
| Completeness (%) | 93.53 (81.64) |
| Wilson B-factor (Å^2^) | 16.71 |
| **Refinement** | |
| Total Reflections | 108186 (11513) |
| Unique Reflections | 56403 (5152) |
| *R*_work_/*R*_free_ | 0.1947/0.2252 |
| Number of atoms: | |
| Protein | 5765 |
| Ligands | 46 |
| Water | 892 |
| Average B-factor (Å^2^) | 25.54 |
| Protein ADP (Å^2^) | 24.78 |
| Ligands (Å^2^) | 10.71 |
| Water | 31.24 |
| Ramachandran plot: | |
| Favored/Allowed (%) | 96.8/3.0 |
| Root-Mean-Square-Deviation: | |
| Bond lengths (Å) | 0.008 |
| Bond Angles (°) | 1.25 |
| PDB code | 7VCM |

Statistics for the highest resolution shell are shown in parentheses.
