# Supplementary material for: A sensitive and specific genetically-encoded potassium ion biosensor for in vivo applications across the tree of life
Source: PLoS Biol. 2022 Sep 6;20(9):e3001772. doi: 10.1371/journal.pbio.3001772 (PMC9481166; doi:10.1371/journal.pbio.3001772)
Supplement: S2 Table — (DOCX) [file pbio.3001772.s002.docx]

**Table S2.** **Mutations accumulated during directed evolution.**

| **Variant** | **Mutations** |
| --- | --- |
| 1 | GINKO1 Template |
| **1.2** | E295F, E296A, L297A, E298N |
| **1.3.10** | Y40N, F151S, E295F, E296A, L297A, E298N |
| 1.3.11 | Q211L, E295F, E296A, L297A, E298N |
| 1.3.25 | K235E, E295F, E296A, L297A, E298N |
| **1.5.6** | Y40N, F151S, N152D, E295F, E296A, L297A, E298N |
| **1.5.20** | Y40N, F151S, K171E, V204L, E295F, E296A, L297A, E298N |
| **1.5.21** | Y40N, F151S, G167C, K201R, V261A, A265D, E295F, E296A, L297A, E298N, N299Y |
| **1.5.36** | Y40N, F151S, T205A, K259N, E295F, E296A, L297A, E298N |
| **1.5.38** | K102E, K201R, K235E, E295F, E296A, L297A, E298N, F373Y |
| **1.5.41** | Y40N, F151S, K284R, E295F, E296A, L297A, E298N |
| 1.6.7 | Y40N, F151S, V154A, T205A, K259N, E295F, E296A, L297A, E298N |
| 1.6.8 | K102E, D130N, I187F, K201R, K235E, E295F, E296A, L297A, E298N,F373Y |
| **1.6.15** | S31Y, K102E, T205A, K259N, E295F, E296A, L297A, E298N |
| 1.6.40 | V2A, Y40N, D130G, F151S, N152D, E295F, E296A, L297A, E298N, E385V |
| **1.7.7** | S31Y, K102E, T205A, K259N, E295F, E296A, L297A, E298N, K356R |
| 1.7.8 | S31Y, K102E, N194D, T205A, K259N, E295F, E296A, L297A, E298N |
| **1.9.8** | S31Y, I94T, K102E, T205A, N266D, K259N, E295F, E296A, L297A, E298N, K356R |
| 1.9.13 | S31Y, K102E, T205A, K259N, E295F, E296A, L297A, E298N, K356R, M383R |
| 1.9.15 | S31Y, K102E, D173V, Q174L, T205A, K259N, E295F, E296A, L297A, E298N, K303Q, K356R |
| 1.9.20 | S31Y, K102E, N183H,T205A, K259N, E295F, E296A, L297A, E298N, K312R, K356R |
| 2.0.17 | S31Y, I94T, K102E, T205A, T241A, N266D, K259N, E295F, E296A, L297A, E298N, K356R |
| **2.0.29** | S31Y, I94T, K102E, N183H, T205A, N266D, K259N, E295F, E296A, L297A, E298N, K356R |
| **2.1.5** | S31Y, I94T, K102E, K127E, N183H, T205A, N266D, K259N, E295Y, E296A, L297A, E298N, K356R |
| 2.2.25  (***GINKO2***) | S31Y, I94T, K102E, K127E, N183H, Q179R, I197V, T205A, N266D, K259N, E295Y, E296A, L297A, E298N, Q307E, K356R, K388M |

The mutations in the template are in black and the new mutations are in red. The variants in bold were used as templates for the next round of directed evolutions. The selected variants in Library 1.4 failed to yield better variants than GINKO1.3.10 and were mixed as templates for Library 1.5. Similarly, a mix of GINKO1.5 variants was used as the template to generate Library 1.6.
