# Supplementary material for: A sensitive and specific genetically-encoded potassium ion biosensor for in vivo applications across the tree of life
Source: PLoS Biol. 2022 Sep 6;20(9):e3001772. doi: 10.1371/journal.pbio.3001772 (PMC9481166; doi:10.1371/journal.pbio.3001772)
Supplement: S3 Table — (DOCX) [file pbio.3001772.s003.docx]

**Table S3**. **Summary of GINKO1 and GINKO2 photophysical characteristics.**

| Name | | Extinction coefficient, EC (M^-1^ cm^-1^) | Quantum yield, QY | Brightness (mM^-1^cm^-1^) | *K*_d_ (mM) | n_H_ | *k*_on_  (mM^-1^s^-1^) | *k*_off_ (s^-1^) | p*K*_a_ |
| --- | --- | --- | --- | --- | --- | --- | --- | --- | --- |
| GINKO1 | - K^+^ | 10000 | 0.42 | 4.2 | 0.42 ± 0.03 | 1.05 ± 0.03 | 9.3 ± 0.3 | 86 ± 2 | 7.9 ± 0.1 |
|  | + K^+^ | 21000 | 0.41 | 8.6 |  |  |  |  | 7.4 ± 0.0 |
| GINKO2  (460 nm excitation) | - K^+^ | 3700 | 0.25 | 0.93 | 15.3 ± 1.1 | 0.91 ± 0.04 | 30 ± 2 | 7.6 ± 0.2 | 7.6 ± 0.1 |
|  | + K^+^ | 37000 | 0.42 | 16 |  |  |  |  | 6.8 ± 0.0 |
| GINKO2 (400 nm excitation) | - K^+^ | 35000 | 0.44 | 16 |  |  | N.D | N.D. | 6.1 ± 0.0 |
|  | + K^+^ | 26000 | 0.31 | 7.9 |  |  | N.D | N.D | 6.3 ± 0.0 |

The extinction coefficient and the quantum yield of K^+^-bound GINKO variants were measured in 150 mM KCl. The values of *K*_d_, n_H_, *k*_on_, and *k*_off_ for GINKO1 were from Shen *et al* (2019)[[13]](https://paperpile.com/c/H557mK/YYTuU).N.D. not determined. n = 3 for *K*_d_, n_H_, *k*_on_, *k*_off_, and p*K*_a_.
