# Supplementary material for: A sensitive and specific genetically-encoded potassium ion biosensor for in vivo applications across the tree of life
Source: PLoS Biol. 2022 Sep 6;20(9):e3001772. doi: 10.1371/journal.pbio.3001772 (PMC9481166; doi:10.1371/journal.pbio.3001772)
Supplement: S4 Table — (DOCX) [file pbio.3001772.s004.docx]

**Table S4**. **GINKO2 two-photon characteristics.**

| Name | | 2P cross section σ_2_ (GM),  λ_max_ in parentheses | 2P brightness F_2_ (GM),  λ_max_ in parentheses | Δ*F*/*F*_0_ (2P) |
| --- | --- | --- | --- | --- |
| GINKO2 | - K^+^ | 14 (948 nm) | 0.28 ± 0.04 (948 nm) | 15 ± 3 |
|  | + K^+^ | 24 (936 nm) | 4.1 ± 0.6 (936 nm) |  |
