# Supplementary material for: A sensitive and specific genetically-encoded potassium ion biosensor for in vivo applications across the tree of life
Source: PLoS Biol. 2022 Sep 6;20(9):e3001772. doi: 10.1371/journal.pbio.3001772 (PMC9481166; doi:10.1371/journal.pbio.3001772)
Supplement: Attachment — Submitted filename: GINKO2 Response to Reviewers .pdf [file pbio.3001772.s021.pdf]

We thank the editor for sending out our manuscript for peer review. We appreciate the positive and constructive feedback from the reviewers. We now have thoroughly considered these comments and carefully addressed the concerns from the reviewers. Please find the following point-to-point response to the reviewer's comments. We have revised the manuscript accordingly. All changes in the manuscript file are highlighted.

Reviewer #1: Potassium ions (K+) are essential for cellular functions across all organisms. In this manuscript, Wu et al. developed a sensitive genetically-encoded sensor to monitor K+ dynamics. Using structure-guided optimization and iterative evolution, a second generation of K+ sensor, namely GINKO2, was developed. GINKO2 shows significantly improved dynamic range, with maximum dF/F0 over 1500%. GINKO2 exhibits excitation ratiometric properties, which provides a convenient way to quantify absolute K+ concentrations across different experiments. Furthermore, GINKO2 is carefully characterized in vitro and is applied into different organisms. In general, the data quality is superb and the control experiments are well performed. The GINKO2 sensor will be a powerful addition to the family of high SNR fluorescent sensors and would help the community in general to investigate the physiological and pathological function of K+. I would support this manuscript to be accepted for publication once the following (mostly) minor issues are addressed.

1. In principle, the genetically encoded fluorescent K+ biosensors have the ability to report K+ dynamics with good spatial and temporal resolution. Unfortunately, this current manuscript falls in short in presenting clear examples of K+ dynamics within the context of good spatial resolution. For example, in Figure 5, the authors only show the expression pattern and time-series curves, yet the spatial difference of GINKO2 across different time points were missing. Similar cases can be seen in Figure 5 and Figure 6.
We thank the reviewer for this valuable suggestion. We have made changes accordingly to emphasize the spatial aspect in the imaging. We have added a new image series (**Figure S11**), which was extracted from **Movie S3** to show the spatial change of fluorescence during CSD propagation. We would also like to mention that the spatial aspect of the GINKO2 response in plants is evident in the kymograph in **Figure S10**.

2. 2-photon microscopy has been widely used with fluorescent sensors to enable deeper tissue imaging in living animals. It would be good for the authors to provide info regarding 2-photon spectrum of GINKO2.
We agree with the reviewer's comments regarding 2-photon. We now have characterized GINKO2 under 2-photon excitation. The 2-photon spectra were added as **Figure 3C**, and key parameters have been added as **Supplementary Table 4.** To explain the results in the text, we added the following sentence: "*The ratiometric excitation is also observed in two-photon (2P) characterization with the maximum fold change of 8.1 at the 2P excitation wavelength of 960 nm (**Figure 3C**)...The 2P brightness of GINKO2 is 4.1 ± 0.6 GM in the K^+-bound state (**Table S4**)*."

3. To image extracellular K+ dynamics in mice, GINKO2 is delivered to the brain by injection of purified GINKO2 proteins. Such a strategy is OK for imaging extracellular K+. This injection is challenging to apply compared with widely used virus-mediated expression. Have the authors tried the viral-mediated method? The authors should clarify or discuss this point.
We thank the reviewer for mentioning this. We have tried to express extracellularly displayed and fully functional GINKO2, but we have been unsuccessful. To clarify this point to the readers, we now have

added the following sentence: "*As previously reported for Kbp-based K^+ biosensor GEPII [36], we have been unable to express and display functional GINKO2 on the extracellular membrane for reasons that remain unclear to us. To circumvent this limitation, we turned to the exogenous application of bacterially-expressed GINKO2 as an alternative method to evaluate extracellular K^+ concentration dynamics during CSD.*" to the section of in vivo imaging in mice.

4. In Figure 2C, the author showed the linker1 and linker2 sequence with two amino acids highlighted in blue and purple, respectively. It is not clear what it means. Figure 2B is similar to Figure1A; thus, I think it will be better to highlight the linker region in Figure 1A. An overall structure with all mutations marked could be used to help the readers to understand the possible function of other mutations that have not been discussed.

We thank the reviewer's suggestions regarding the structure figures. To explain the colour-coding in **Figure 2C**, We have added "*Green-coloured residues are on GFP, orange-coloured residues are on Kbp, grey coloured residues are on linkers, and blue-coloured L and magenta-coloured E (N in GINKO1.2) are the positions of 'gatepost' residues that define the insertion points in EGFP*" in the figure legend. We have also changed the linker2 colouring in **Figure 2B** to match the colour-coding. Following the reviewer's suggestions, we added an overall structure with mutation residue numbers labeled and highlighted in **Figure S2E** and **Figure S2F**.

5. In Figure 1 legend, I believe the structure is for "GINKO1", which may be mistakenly labelled as "GINKO2".

We thank the reviewer for pointing out this typo. The figure title has been corrected.

6. In Figure 4, it would be good for the authors to provide a cartoon to illustrate how the experiments are performed. The similar case in Figure 7.

We have followed the reviewer's suggestion and added **Figure 4A** to explain the bacteria experiment and **Figure 7A** to show the preparation of the *Drosophila* samples for imaging.

7. In Figure 6, the experimental design should be labeled more clearer, e.g., how was GINKO2 delivered? Additionally, the experiments were done in 2 mice? I suggested to do more repeats in different mice.

We have added an explanation in the legend of Figure 6: "*Exogenously expressed GINKO2 protein was purified and externally applied to the imaging site by pipetting*." The experiment was performed in two mice. For each mouse, there were five technical replicates. All the replicates showed consistent results.

Reviewer #2: PBIOLOGY-D-22-00474R1 Wu et al.

This manuscript reports the structure and characterization of a genetically encoded potassium sensor, GINKO1, based on the insertion of the E. coli potassium binding protein Kbp into a circularly permuted version of green fluorescent protein (GFP). The structural information is used for a mutant screen to identify an improved version, GINKO2, which is then tested in a variety of systems including bacteria, plants, and mice. Although the structure is interesting, the quality of the work and presentation is serious flawed. Moreover, the fundamental question of how a sensor having a reported Kd for potassium of 15 mM, which is at least an order of magnitude below the concentration of intracellular potassium can accurately report on potassium concentration changes is never addressed in a quantiative way. This fundamental quantitative mismatch is never adequately addressed. Moreover, GINKO2 has a sensitivity to pH. Hence one is left wondering what parameter this sensor actually reports on in cellular contexts.

We appreciate the reviewer's concern regarding the K_d value of GINKO2. We would like to point out that the K_d of 15 mM is measured *in vitro* with purified protein, which is not necessarily the same as the K_d in the cellular context. We have performed *in situ* titrations of expressed GINKO2 in permeabilized HeLa cells (**Figure 3I**), *E. coli* (**Figure 4C)**, and plant cells (**Figure 5B**). These results all demonstrated highly dynamic fluorescence changes of the sensor in the physiological-relevant K^+ concentration range, suggesting its compatibility with intracellular K^+ sensing. The K_d values in the cellular context were not reported because they can not be reasonably estimated due to the lack of an upper plateau.

Despite the very high resolution data for the structure (1.85Å) the analysis of the potassium binding site is surprisingly superficial. No distances are given for the potassium coordinating carbonyl backbone ligands (Fig. 1D). This is the most critical parameter for understanding how Kdp might serve as a potassium binder.

Following the reviewer's suggestion, we have added the key distance measurement in the K^+ binding site **Figure 1C**.

Given the unusual nature of the binding site, it is surprising that there is no comparison to other well-characterized protein based potassium binding sites (channels, Na+/K+ ATPase, for example), all of which use an apparently similar strategy in which backbone carbonyls coordinate the ion. There is also no comparison to the well studied potassium binding circular peptide valinomycin, which similarly coordinates potassium through interactions with carbonyls.

We have compared the K^+ binding site of GINKO1 to K^+ channels KcsA and TrkH with the following text: "*Notably, the K^+ ion is coordinated via six backbone carbonyl oxygen atoms (from amino acids V154, K155, A157, G222, I224, and I227). This coordination via backbone carbonyl oxygen atoms is similar to that observed in the K^+ selective filters of KcsA (PDB ID: 1BL8)[15] and TrkH (PDB ID: 4J9U)[16], as well as K^+-coordinating compound valinomycin[17]. The distances of coordinating carbonyl oxygens to K^+ in GINKO1 range from 2.6 to 3.2 Å with a mean value of 2.8 Å (**Figure 1C**), similar to those in KscA (2.70 – 3.08 Å, with a mean value of 2.85 Å)[18], valinomycin (2.74 – 2.85 Å)[17]. One difference is that K^+ is coordinated via eight backbone carbonyls in both KcsA and TrkH, and six backbone carbonyls in Kbp.*"

The figure showing comparison with the isolated Kbp structure is poor quality. Although potassium was not located in that structure, it was determined under conditions in which the potassium binding site

should be occupied. Are there meaningful changes between the crystal structure here and the Kbp structure? RMSD values are missing.
We have replaced **Figure 1D** with a superimposition of the isolated Kbp domain in GINKO1 and the original Kbp structures. We also replaced **Figure 1E** with a zoom-in view of the binding pocket in the GINKO1 crystal structure and the Kbp solution structure. RMSD values are provided in **Table S1**.

Fig. 1 also lacks a cartoon showing the design of GINKO2. Even though the design for GINKO1 has been published before, showing such a diagram is essential for understanding how and where Kbp is inserted into the circularly permuted GFP.
We thank the reviewer for the suggestion. We have added **Figure 1A** to show the cartoon of GINKO sensor design.

Figure S1 is entirely uninformative and has poor quality data. The SEC is overloaded and provides no information about the molecular size of the protein and cannot be used to support the claim that GINKO1 is a monomer. There are no molecular weight standards. The peak shape is exceptionally broad and provides no information about the molecular size at it appears to span most of the included volume of the column. There is no reason to show the diffraction pattern. It is striking, given the claimed resolution of 1.85Å, that there are no figures showing exemplar electron density. Such information is absolutely essential for supporting the claims. Density of the putative potassium binding site should be shown.
We agree with the reviewer's comments. We now have removed the SEC data in **Figure S1A** and the diffraction pattern in **Figure S1B**. We also added the electron density map of the GINKO1 ligand binding site as the new **Figure S1B.**

Fig. 2 is entirely uninformative as it only shows a portion of the interface where the mutations are made. None of the context of the interactions of the linker residues is shown, making it impossible to understand how the mutations may be acting.
We appreciate the reviewer's comments on **Figure 2. Figure 2 is** meant to illustrate the engineering process of GINKO2. Panel A-C are graphed to better visualize the locations of structure-guided mutations in the GINKO1 structure. **Figure 2A** highlights the position of chromophore-interacting residue E295. **Figure 2B** highlights the linker region between the EGFP and Kbp. **Figure 2C** informs detailed sequence changes in overall sensor design. Panel D is graphed to illustrate the improvement of the sensor performance through directed evolution. Overall, we believe that this figure is informative and instructive for the readers, especially from a protein engineering perspective. To help the readers better understand how the mutations might be influencing the function, we have a dedicated paragraph discussing some of the beneficial mutations. These mutations are illustrated in **Figure S4 A-D.**

The text says that the linker was randomized for the selection experiments to improve GINKO. However, the methods simply state that random mutagenesis was used. It would appear that this covers the entire protein, not the linker.
We thank the reviewer for pointing this out. To clarify this, we have now edited the sentence as follows: "*...we performed site-directed saturation mutagenesis on the linker residues connecting EGFP to Kbp…*"

The selection of clones with new properties is uncelar. E coli has 250 mM K+ in the cytoplasm (Weiden J Gen Physiol 50:1641-1661m1967). Hence, by searching for brighter GINKO variants, it is unclear exactly what the selection is based on.

To better explain the selection of new variants, we now have added the following paragraph to the legend of **Figure S3:** *"Variants were preliminarily screened based on the colony brightness because high fluorescence intensity of a variant in bacterial cytosol (a high K^+ environment) could correlate to a high brightness in the K^+-bound state, which is desirable for a positive-response biosensor…. In the secondary screening, variants with the largest fluorescence changes were selected for further characterization and winning variants were used as templates for the next iterative round of evolution.."*

The parent Kbp has a reported affinity for potassium of 160 µM. Given the structural data, is there any way to rationalize how the affinity has been lowered in the GINKO constructs?

We appreciate the reviewer for mentioning this. Since we only have the crystal structure of GINKO1 in the K^+-bound form, there is no sufficient structural information to rationalize the change in the affinity.

The structure is potentially interesting. The potassium responses are unconvincing given the quantitative considerations.

We are unsure how to interpret this comment. We would appreciate it if the reviewer could provide clarification regarding which quantitative considerations they are referring to, and exactly what is unconvincing about the potassium responses.

Reviewer #3: Wu et al present the crystal structure of GINKO1, the first generation of a K+ biosensor. Based on the structural information they engineered GINKO2, a massively improved, ratiometric K+ sensor, which was shown to be applicable in bacteria, plant cells and in Drosophila, as well as extracellularly applied to mice.
The data presented in the manuscript are very conclusive and GINKO2 will provide a valuable tool for studying K+ homeostasis in cells and tissues. The sensitivity and selectivity of GINKO2 is much improved when compared to GINKO1.

I have one major criticism that could easily be addressed. The ratiometric behavior of GINKO2 has only been shown for purified protein and is then used as calibration curve for in vivo experiments. It would be important to show a similar ratiometric behavior of GINKO2 in cells: One possibility would be valinomycin- and protonophore-permeabilized E. coli cell incubated at a neutral pH but with changing K+ concentrations. This way the intracellular potassium concentrations would equal the extracellular concentrations while having GINKO2 in a cellular environment.
We appreciate the reviewer's valuable comments. We have now followed this advice and performed such an experiment. The calibration curve obtained with valinomycin-treated *E. coli* cells has been added in **Figure 4C**.

Additionally, I have some smaller criticism:
1. The review that summarizes the role of potassium homeostasis in bacteria is a bit outdated. In recent years a lot of progress has been made with respect to bacterial potassium homeostasis, which actually better highlights the need of good K+ biosensors. I suggest to cite one of the more recent reviews or actual primary literature.
We followed the reviewer's suggestion and added the following review to the reference: *Beagle, S. D. & Lockless, S. W. Unappreciated Roles for K+ Channels in Bacterial Physiology. Trends Microbiol.* ***29,*** *942–950 (2021).*

2. You state 'Notably, the backbone carbonyl oxygen atoms of six amino acids (V154, K155, A157, G222, I224, and I227) coordinate K+, similar to the coordination sphere of K+ selectivity filters in K+ channel KcsA and K+ transport protein TrkH [14].' In fact in both ion channels the potassium ion(s) in the selectivity filter are coordinated by eight not six carbonyl oxygen atoms. This might also explain the higher affinity of these proteins for potassium ions. Note that also TrkH is a potassium channel not a transporter. Also, your citation is not appropriate. The selectivity filter of TrkH is not even shown in that review. I suggest you rather cite primary literature!
We thank the reviewer for pointing this out. We have now added the primary literature citations for the TrkH and KcsA structures. We have now also mentioned the difference in the number of coordinating carbonyl atoms. However, we ultimately decided that it was not appropriate for us to comment on how this might affect the relative affinity for K^+, as the role of channels is selective permeability to K^+, and Kbp is simply a binder of K+, we are not sure that it would be possible to meaningfully compare the binding sites in terms of affinity and specificity. "*Notably, the K^+ ion is coordinated via six backbone carbonyl oxygen atoms (from amino acids V154, K155, A157, G222, I224, and I227). This coordination via backbone carbonyl oxygen atoms is similar to that observed in the K^+ selective filters of KcsA (PDB ID: 1BL8)[15] and TrkH (PDB ID: 4J9U)[16], as well as K^+-coordinating compound valinomycin[17]. The distances of coordinating carbonyl oxygens to K^+ in GINKO1 range from 2.6 to 3.2 Å with a mean value of 2.8 Å (**Figure 1C**), similar to those in KscA (2.70 – 3.08 Å, with a mean value of 2.85 Å)[18],*

valinomycin (2.74 – 2.85 Å)[17]. One difference is that K^+ is coordinated via eight backbone carbonyls in both KcsA and TrkH, and six backbone carbonyls in Kbp. "

3. Sentence 'This mutation may help to stabilize the K + -bound GINKO1? by reducing the distance to D148 and hence increasing their electrostatic interaction' on page 5 is somehow wrong. Why '?' and are you really referring to GINKO1 here?

We thank the reviewer for pointing out this typo. "?" should not have been there, and we have deleted it.

4. Why would GINKO2 not respond to Na+? Any speculation based on the structure?

We thank the reviewer for mentioning this. We now added a speculative explanation to the maintext: "*As the affinity for K^+ of GINKO2 (15.3 mM) is substantially lower than that of GINKO1 (0.42 mM) (**Table S3**), the affinity for Na^+ may have also decreased proportionally. Since the K_d value for Na^+ of GINKO1 is 153 mM, a proportionally increased Na^+ K_d in GINKO2 would be well outside of the physiologically relevant range of Na^+ concentrations."* We have not attempted to model other ions in the binding site, and so we do not have any structure-based insight into the specificity of the binding site.

5. Several panels of different figures appear to lack error bars although the authors state at least triplicates have been performed.

Some errors were hardly seen because the S.D. values were too small to show. Examples include some data points in **Fig 3D–G, 4C, 5C (lower panel), S5, and S6**.

6. Fig. S6 correct RuCl to RbCl

We have changed RuCl to RbCl in Figure S6.
